# What should AI explain to you? Visions of older adults regarding explainable AI

**DOI:** 10.1093/geront/gnaf308

**Published:** 2026-02-03

**Authors:** Catharina Margaretha van Leersum

**Affiliations:** Faculty of Humanities, Open Universiteit, Heerlen, The Netherlands

**Keywords:** Age in place, Gerontechnology, Decision-making, Black box

## Abstract

**Background and objectives:**

Artificial intelligence (AI) combined with smart sensors or conversational agents are becoming part of our lives, and have the potential to improve aging in place by supporting independent living. Trust and willingness to use AI seem essential for actual embedding. Explainable AI (XAI), originating from the recognition that AI infrastructures often operate in an opaque, “black boxed” way, might assist in understanding the underlying logic of AI-made decisions. However, it is unknown what older adults think about XAI and what they consider as needed explainability.

**Research design and methods:**

I conducted 28 semi-structured interviews to explore XAI in the worlds of older adults. Inductive analysis was applied to analyze what do older adults know about AI and how do they imagine our society with AI? What is XAI to them and how do they value explainability?

**Findings:**

The analysis resulted in nine themes: four regarding the knowledge on AI of older adults, including definitions, knowledge acquisition, attitudes, and expectations, and five themes were leading for the views on XAI in which XAI is not desirable, XAI is necessary, or collaboration is preferred.

**Discussion and implications:**

The visions of XAI are different from current technological discourses. For older adults, XAI is not only technological, but a constellation between humans and machines. Most argue that a form of joint decision-making is important. As a follow-up, it seems recommended to explore the enactment of XAI in real life, and investigate the form or degree of XAI needed and for whom.

## Introduction

The number of older adults will increase in the coming years (Chen et al., 2023). For this reason, the policy of different countries, among others the Dutch health policy, aims to allow older adults to remain living at home as long as possible. With this expanding number of older adults living independently, the goal to support healthy aging in place seems crucial. While aging, assistance is often provided by family, and technologies can bring some support in performing daily activities to help individuals remain autonomous and safe at home (Calvaresi et al., 2017). In this view, I talk about gerontechnologies, which are technologies specifically designed for older adults (Kwon, 2016). The introduction of these gerontechnologies impact the care practices of family members. This impact is varying, because on one hand, a family member can be relieved from certain tasks and the relationship between family members becomes restored (van Leersum et al., 2023), but on the other hand, technology can be seen as an intruder in the relationship between family members (van Leersum et al., 2024).

### Artificial intelligence as gerontechnology

In this study, I will focus on the visions of older adults, living at home, regarding artificial intelligence (AI) and the need for explainability of AI, because, recently, attention in gerontechnology development has shifted toward AI and its potential to support aging in place, and provide “better care for older people” and “the right care at the right place” (Chen, 2018; ­Gallistl, Banday, et al., 2024; Timans et al., 2024). The development of AI systems as gerontechnologies started in the 1990s, mainly including monitoring and automated alarm systems to support the aging population (Chan et al., 1998; Gallistl, von Laufenberg, & Lehner, 2024). Currently, AI systems are being tailored to current living conditions of older adults and range from monitoring and surveillance to decision support systems, and from automated data analysis to conversational agents (Chen, 2018; Gallistl, von Laufenberg, & Lehner, 2024; Liu et al., 2020; Sapci & Sapci, 2019; van Leersum et al., 2023).

AI is introduced to support aging in place, and it is becoming part of our lives, among others smart watches, virtual reality, chatbots, and writing support (Tielman et al., 2024). Besides all the support AI might provide, current literature discussed multiple ethical issues such as responsibility, data protection, fairness, diversity, safety, autonomy, human agency, and trust (van Leersum & Maathuis, 2025). Most of these ethical issues are related to the fact that AI systems are considered as “black boxes”. The image of AI as a black box means that the internal mechanisms and reasoning of the AI are often unknown for the user. Here I refer to the technological complexity of AI stemming from algorithmic processes based on large amounts of data and how AI is mystified to detect patterns beyond human intelligence (Campolo & Crawford, 2020).

### Explainable AI

Several authors argue that low trust and low understanding of the reasoning behind AI systems are the main reasons for low willingness to use AI (Elish, 2018; Meske et al., 2022; Zerilli et al., 2022). To enhance willingness toward use, hope is currently pinned on explainable AI (XAI). The research field of XAI originates from the objective to tackle the black boxing of AI. In this research field, many focus on the development of mathematical methods to make AI-made decisions pre- and post-modelling explainable (a.o. Adadi & Berrada, 2018; Angelov et al., 2021; Arrieta et al., 2020; Minh et al., 2022; Tjoa & Guan, 2021). Their goal is to create XAI to assist users in understanding the underlying logic of the decision-making process, and give them the possibility to identify mistakes, turning an opaque black box into an interpretable twin “white box” (de Fine Licht & de Fine Licht, 2020). Although an uptake in the social sciences is required according to the engineers working in the XAI field, there is little known about user experiences of current explanation methods (Butz et al., 2022), and there is scant empirical research on the topic of XAI.

### Engineering or humane XAI

In view of research on XAI, computer scientist Tim Miller argues that creating an AI system explaining itself and its decision-making process seems impossible. To underline this statement, Miller (2019a) uses a quote from AI researcher Geoff Hinton, who argues that people also cannot explain how they work because decisions are based on all sorts of unquantifiable things, and to explain someone has to make up a story. Still, we can learn from looking at people and investigate how people explain to each other, what kind of questions they ask, how they answer these questions, and how do they determine whether someone understood the explanation (Miller, 2019b). Only a few XAI methods describe how they can be presented to end users, but most do not imitate human reasoning (Butz et al., 2022). This shows that current XAI developments are based on intuitions of AI engineers and experts, which can feel daunting and offer little insight to nonexperts (Butz et al., 2022; Miller, 2019a, 2019b). Combining these intuitions with a human manner of explaining can form a starting point and the basis of explainability.

This is acknowledged by Shortliffe and Sepúlveda (2018) who argue that AI systems need to provide a form of explainability to enhance human understanding, and the delivery of information should acknowledge the expertise of users. Many users will probably not understand algorithmic logic and need a different type of explanation, which connects to questions raised by Tielman et al. (2024) regarding the level of understanding reachable by users and how explainable a system should be. Also Elish (2018) argued that there is an important distinction between showing “that” a system works and showing “how” it works. There is ongoing research to make the systems technically interpretable, but this does not mean that it will be understandable or trusted by different users (Elish, 2018), mainly because there is a lack of consensus around explainability (Miller, 2019b).

Thus, besides all attention on creating mathematical methods to make AI more explainable, we should combine this with a focus on people. There is an interconnectedness of the technical dimension of an AI system and the social/human dimension of beliefs and context in which the tool interacts (Sendak et al., 2020). With this sociotechnical systems perspective, Sendak et al. (2020) demonstrate that explainability is not enough to ensure trust, accountability, and transparency. Explainability is not an end in itself, but enacted in a broader social context and needs of people (Kluttz et al., 2022). Therefore, Green and Chen (2019a) argue that XAI should be designed as an “algorithm-in-the-loop” to place AI-made decisions within a sociotechnical view to improve human decision-making rather than focusing on the technical context solely (Green & Chen, 2019a; Pesapane et al., 2024). With the term algorithm-in-the-loop, Green and Chen (2019a, 2019b) introduce the attention on human–algorithm interactions. In their view, these interactions should improve human decision-making instead of using humans to improve algorithms. In care settings, for example, a physician has to interact with an AI machine. The physician will be informed by receiving an outcome. Then due to the interaction, the AI and physician make the decision in collaboration. However, in this theory, it is still unknown how algorithms and humans interact (Green & Chen, 2019b).

### Co-constitution of aging and AI

Taking from the above, explanations need to be developed and shaped in relation to the users and their context. This connects with the co-constitution of aging and AI (Gallistl, Banday, et al., 2024), which contains a set of practices to reflect on the relationship between aging and AI. This theory is based on the co-constitution of aging and technology (CAT) by Peine and Neven (2019). CAT highlights how aging and technology mutually shape each other and come into being in relation to each other (Peine & Neven, 2021). To empirically understand what it means to age with technologies, aging and technology can be studied where they arise together in the design-world and life-world. Gallistl, Banday, et al. (2024) developed this further into the co-constitution of aging and AI. Their description highlights four sets of practices: living with AI, practices of datafication, designing AI, and contextualizing AI. It is recommended by the authors to reflect on how the relationship between aging and AI might be imagined, to explore how AI systems are contextualized and embedded in everyday activities, and how these arrangements and activities are changing due to the embedding of AI (Gallistl, Banday, et al., 2024). I will use these ideas in my research to design the interviews and explore the visions and contextualization of AI according to older adults, and thus how aging and AI are intertwined and formed in the design-world of AI and life-world of older adults.

### Objective

In my earlier work, I proposed a layered XAI model that suits different stakeholders with different needs for XAI (van Leersum & Peine, 2025). This is in line with recent research of Tielman et al. (2024) on “explainable AI for all.” In this work, they argue that existing literature does not provide a definition for XAI. Therefore, XAI can consist of any type of information conveyed by an AI system to a user about what it does, how it does that, and why, including both inner working mechanisms and processes of how a system was made (Tielman et al., 2024). XAI should be targeted toward the user or other relevant stakeholders, but I also argue that even within the same context or user population the need for XAI might differ and different forms of XAI are necessary.

Although different stakeholders were included in my previous research, older adults were not yet included. There are some studies investigating the attitudes of older adults toward AI. Wong et al. (2025) show that most acknowledge potential benefits of AI in the care domain, but AI should not replace human interaction. Older adults are open to learn about AI, but hesitating due to worries regarding lack of leaning abilities (Shandilya & Fan, 2024). Furthermore, most empirical research applies quantitative methods focusing on the usability of AI. They show that usability is influenced by personality traits (Shade et al., 2025) and older adults have reservations to follow AI instructions (Vondenberg et al., 2024). However, to the best of my knowledge, there is no empirical research involving older adults or any other/younger non-AI expert to explore the meaning of XAI.

I will make a start in researching this gap by exploring what XAI means in the life-worlds of older adults. Furthermore, I contribute to the existing knowledge on AI attitudes and knowledge or use by older adults. What do they know about AI and how do they experience and imagine our society with AI? What is XAI to them and how do they value explainability? Answering these questions is interesting in view of future development and embedding of AI, and designing XAI for the aging population.

## Research design and methods

I applied qualitative descriptive research to explore what older adults know about AI and what they consider as explainable. Semi-structured interviews were conducted to explore XAI in the worlds of older adults, their knowledge about AI, and how they imagine XAI.

### Setting and recruitment

This study was performed with older adults living in their own home—as owners or tenants—in the Netherlands. To recruit the older adults, a purposive sampling strategy was applied. A call for participation was spread among members of three social associations for older adults. To reach a diverse population, I contacted three associations in three distinct areas of the country. Two are based within larger cities (one large and one middle large city), and one was based in the provinces. Together, these associations have almost 3,000 members, all 60 years and older, which represents about 10% of older adults living in the Netherlands. The older adults were invited to call me or send me an email. I contacted all who showed interest to participate to provide more information, and when willing to participate, a meeting was planned.

Twenty-eight participants were included. Thirty-six percent was male and 64% female, aged between 66 and 86 years with an average age of 74 years. Nine had received no or a practice-based educational degree, 13 a degree in applied sciences, and six a university degree. One participant was born in America, one in Indonesia, one in Curacao, and the other 25 had a Dutch nationality. Ten received home care, and all others considered themselves as healthy with normal deterioration due to aging. I also questioned their view toward technology following the innovation adoption curve of Rogers (2003). Based on their personal identification, most older adults identify themselves as the early majority. There were three who saw themselves as innovators and six as laggards of whom one did not possess a smartphone or any device to ­connect to the internet. Table 1 shows all participant characteristics.

**Table 1.** Demographic characteristics of the older adults (*N* = 28).

Characteristics	Number of older adults (%)
**Sex**	
** Male**	10 (36%)
** Female**	18 (64%)
** Other**	0 (0%)
**Age range (years)**	
** 60–69**	5 (18%)
** 70–79**	19 (68%)
** 80–89**	4 (14%)
**Education**	
** None**	2 (7%)
** Practice-based**	7 (25%)
** Applied sciences**	13 (46%)
** University**	6 (21%)
**Living conditions**	
** Home owner**	17 (61%)
** Tenant**	11 (39%)
**Work**	
** Retired**	24 (86%)
** Almost retired**	4 (14%)
**Nationality**	
** Dutch**	25 (89%)
** Non-Dutch**	3 (11%)
**Receives care**	
** Yes**	10 (36%)
** No**	18 (64%)
**Type of innovator (** Rogers, 2003 **)**	
** Innovator**	3 (11%)
** Early adopter**	5 (18%)
** Early majority**	8 (29%)
** Late majority**	6 (21%)
** Laggard**	6 (21%)

### Data collection

Data were collected between June and September 2024. Twenty-eight interviews were conducted to investigate what older adults know about AI, and how they experience and imagine our society with AI. Furthermore, their vision on and how they value explainability was questioned. An interview guide was developed on the basis of literature and my earlier research on XAI (van Leersum & Peine, 2025). The interview guide was discussed with two older adults of the involved social associations before starting the actual interviews. Their advice was to not directly start about AI—since it could be a “frightening” topic—but gradually build toward this topic while talking about technologies. The topics in the guide included technology in general, their knowledge about AI, the role of AI and their visions on our society, what is XAI, and what they think about explainability/need for XAI (full interview protocol in Supplementary material 1). The interview protocol shows that I asked the older adults whether or not they were familiar with terms like AI and XAI, and if they were able to provide a definition. If they were not able to define the terms, I assisted them by giving a definition before continuing the conversation about AI or XAI, respectively.

I asked all participants whether they prefer physical, online, or phone-based interviews. One interview was conducted online with the use of Microsoft Teams, one interview was conducted through a phone call, and 26 interviews were physical one-on-one conversations. All interviews were conducted by the same researcher and lasted 48 to 110 min, with an average of 62 min. Audio-recordings were made of in total more than 29 hr.

### Data analysis

Verbatim transcripts were made from the audio-recording with support of Amberscript software. Data were anonymized to maintain confidentiality. An inductive analysis approach (Elo & Kyngäs, 2008) was applied to all data with the use of software package Atlas.ti. Data saturation was reached when no new themes emerged. As a first step, I read four transcripts and applied codes to all quotes. Second, my initial findings were discussed within the department during monthly meetings. Then, I combined the codes into overarching themes to identify the most prominent ones and develop an initial coding matrix. As a third step, this coding matrix was used to read the other 24 transcripts and codes were accorded to quotes. During this third step, new codes emerged in sections where none of the previous identified themes fitted. A final coding matrix emerged consisting of nine themes based on 374 codes. As a last step, the coding matrix was used to define the structure of the findings, with four themes showing the knowledge and visions of older adults toward AI and five themes regarding visions on XAI.

### Trustworthiness

Credibility was established by several procedures (Lincoln & Guba, 1985). Investigator triangulation was reached by including a second researcher with whom I could discuss the research design, interview findings, and analysis. Peer debriefing took place at monthly meetings within my department during which the scientific and organizational aspects of research were discussed. A member check was done by sharing a summarizing document of the interview data with all older adults and asking them to recommend, and a presentation to show findings was organized at the three social associations. Transferability was approached by a thick description (Lincoln & Guba, 1985), which included the recruitment approach, participant information, interviewing method and developing of materials, data collection, and analysis process. This research could be seen as transferable to other contexts of aging, such as institutional care, to different countries, to different age groups including younger people, and to specific AI systems.

### Ethics

Ethical review and approval were obtained from the Research Ethics Committee of the Open University (reference number: Ceto_RP559). The participants received an information letter, were informed about their right to withdraw at any time, and informed consent was obtained before the interviews. Data were anonymized and data confidentiality was maintained.

## Findings

**Figure 1.**
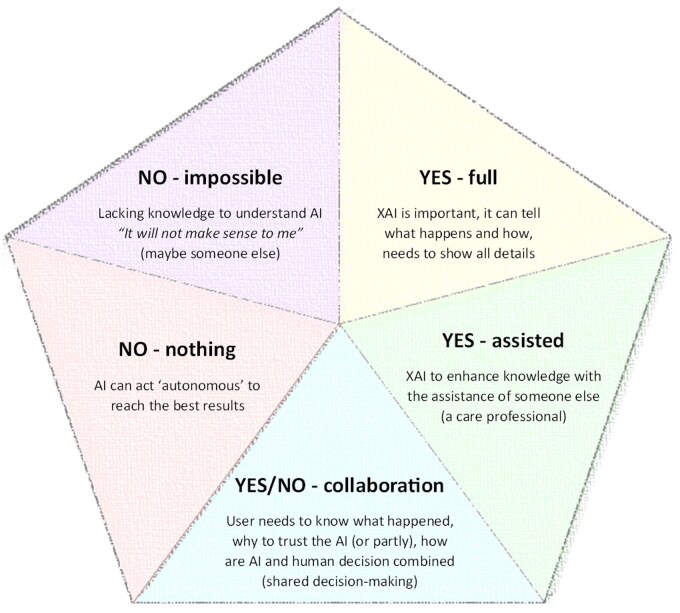
Five views toward XAI as envisioned by the older adults. AI = Artificial Intelligence; XAI = Explainable AI.

The large number of participants with a diverse affinity of technology enabled me to provide a wide perspective on the visions of older adults toward AI and XAI. The analysis of all 28 interviews resulted in nine themes: four themes regarding knowledge on AI, and five themes on XAI. First, I present the findings regarding the attitude and knowledge on AI by the aging population. This includes (1) the definitions they provide to AI, (2) how they acquired knowledge, (3) their attitudes toward AI, and (4) their expectations regarding AI. Second, I present how the older adults envision XAI in our society and their lives. Five themes were identified that lead into views toward XAI (Figure 1), in which two themes are regarding desirability, two in line with necessity, and the last one on collaboration to reach XAI.

### Knowledge on AI

To start these findings, I explored what older adults know about AI. In the worlds of the older adults, they experience being part of a transition from mechanical toward digital, which is received with more or less enthusiasm. I spoke about this transition with all older adults, and most acknowledge the change and that they are not able to ignore it. With the exception of one participant, all are using one or more digital devices. However, they are not all welcoming the changes and with the introduction of AI it becomes even more alien and different from what they know. In this first section of the findings, I will discuss how older adults define AI, acquire knowledge about AI, what their attitude is toward AI, and what they expect from AI.

#### Ideas about the definition of AI

All the older adults have heard about AI, but when I asked them to provide a definition they all struggled. They reason that AI is new for the aging population and that they never learned anything about this kind of technology; therefore, they are hesitating to adopt and learn about it. Before giving them a definition and examples of AI, a common element almost all mention is the idea that AI contains information to become “as smart as or even smarter than a human being.” A small group knows a bit more about AI and adds to this part that they are only smarter in view of a specific topic, for example, playing a game like chess. However, most argue that the systems they are aware of are not really smarter than a human, but try to take over certain tasks. They mention the use of chatbots by certain companies to answer questions of customers:

You can use them to ask the really, really, really easy questions, but for anything else it is impossible to get a useful answer. Sometimes it is mandatory to start a request, for example to get help with an online order, trough such a chatbot. So, I started typing the question. But instead of getting an answer you get the remark ‘please rephrase your question, I do not understand’. Or it asks me all kind of questions first which I need to answer before it can be of any help. For me, this is not helpful at all! (Older Adult 5)

None of the older adults who tried out chatbots were a fan, and all tried to avoid using them because they got the idea that they were never really understood and still needed to consult a human employee.

Overall, when it comes to the definition the older adults provide, AI is aimed to be smarter than a human, learn on the basis of large data sets that we—as society—provide and make, and can take over jobs. At the end of the interviews, most understood that this last vision of taking over jobs can be nuanced, and they felt the need for more knowledge to fully understand what AI might bring in their lives and mean to our society.

#### AI knowledge acquisition

The way most older adults acquire knowledge about AI can explain their idea of taking over jobs as well. When asking how they learned about AI, they tell that they heard or read about it in the news, journals, and talk shows. One person heard about AI a couple of times, but predominantly avoids media channels because the news makes her really sad about our society. She knows a bit about AI from the home care organization who gave her the option to place a form of intelligent sensors in her home. However, she was not a fan of this idea and decided to handle her situation differently:

Sometimes I am scared at home, I feel alone and I not sure if someone will find me when there is anything wrong. My home care provider asked me if I wanted some kind of security devices around the house. They explained that these consisted of sensors that could detect any form of movement. I have no idea how it works, but it sounded like something valuable for my well-being. Thus, I asked them to instal the sensors. Then, I had to instal some kind of app, which I really did not like. A couple of days ago, when I was watching this talk show, they warned people about AI due to all kind of privacy issues. Then, also someone else had to instal this app to get messages about my movements. I did not want to reject the sensors immediately since she just helped me with everything. But after three days, one of daughters texted me if everything was ok. I was not detected in the living room that morning… It scared the hell out of me, I left my home early to meet a friend and indeed did not enter the living room. I directly asked my care provider to remove all. Now, every morning I send a message to my daughters and then they know I am alive. (Older Adult 20)

Except for three, all believe in the truth of messages provided through media channels. The three who are skeptical toward these messages explained how they try to determine the reliability of the media platform or author. Although this kind of media is mostly trusted, all participants are skeptical toward social media, which is an unreliable source and meant to divide our society:

It is heartbreaking if you think about the difficulties my grandchildren and their friends have with the current overload of media and information. Social media is the worst and they will read all these messages and rely on them or create suspicions towards everything and towards everyone. That is not a world in which they should grow up and become older. (Older Adult 26)

Multiple older adults share this feeling and they agree that it is much more difficult to know what to rely on nowadays. People need to learn about what to trust and how to cope with all information sources.

A large part is not searching for information about AI themselves, because they argue that it is hard to understand or they have no idea where to search for it. Just a small part actively searches for additional information on AI and the development of new systems to improve their knowledge. One person was a programmer herself and already learned in her education about AI in the early days. But overall, a common way of dealing with knowledge on AI was, “I hear about it and now can only let it happen to me, I cannot do anything about it” (Older Adult 11).

### Attitudes toward AI

The initial attitude and feeling toward AI is often negative. As their knowledge is based on news, journals, and talk shows, it is understandable that quite some have the reaction, “it is frightening because we cannot control it or it will take over the world.” During discussion about AI in care settings, some were anxious that AI would take over the diagnostic process for physicians. For example, Older Adult 8 mentioned,

It could be helpful if AI is capable to diagnose certain diseases. However, I think a physician should always understand what is happening in the AI. Diagnosing dementia sounds like a very difficult case… If the AI systems can detect dementia which is still not detectable by a physician and maybe not even affecting my life, do I want to know it? It sounds like something futuristic, but if you say it might be possible soon, we need to determine how to handle this kind of diagnostic processes. I cannot imagine how many ethical issues are detached to this topic. (Older Adult 8)

This dementia case was discussed with multiple older adults and most agreed that it is not desirable to know everything, especially if a disease is not manifested and not treatable. When it is about early detection of cancer by AI, most were very much in favor of knowing and acting. Furthermore, the overall consensus regarding who is involved in the decision-making process ended in having the physician still as the one conveying diagnosis is crucial, and thereby, the physician needs to make a final decision which diagnose to mention, not the AI system.

When it comes to intelligent machines and digital technologies in general, “it is impossible to fix anything yourself”, which was still possible with mechanical technologies. Older Adult 1 gives the washing machine as an example. Her machine just broke 2 days ago and she could not do anything herself to fix, in contrast to older machines. She was amazed by the mechanics who had to use a tablet to fix the machine externally. Similarly, Older Adults 2 and 18 talked about fixing cars. This was possible and interesting when they were younger, but now even car service stations attach the car to a computer to know whether there is anything wrong, and owners cannot do much themselves.

On the contrary, there are older adults with more positive feelings toward AI and digitalization. According to them, the transition toward a digital society has brought us many good developments and benefits for our way of living. Although our society is changing, not all changes are negative, and it might take time before we fully embrace AI because we have to learn how to use and incorporate it into our lives:

We cannot escape from AI developments, but we should not because they can assist us as aids. For example, to communicate with relatives in case of emergency. If I fall, my watch will message my son. This gives a very reassuring feeling because he is not close. However, I already experienced that the watch ‘detected’ a fall when I did not actually fall. Luckily, I noticed the mistake and was able to notify my watch that I did not fall. I am not sure what would have happened otherwise, probably my son receives a message about my incident and he would worry about me or already send an ambulance to my home. This kind of mistakes need to be minimalized. (Older Adult 4)

Besides some positive attitudes, there is genuine interest in the abilities of AI. One older adult has the vision of AI systems able to take blood samples. In contrast to humans, she thinks that these systems will perform better and will be honest if they are unable to draw blood from her veins. Another older adult envisioned that all humans will have brain implants in the coming years, helping us with difficult tasks, and especially help the aging population to overcome memory problems: “I am looking forward to this future! It would be very helpful for everyone and might overcome brain problems” (Older Adult 9).

#### Expectations and use of AI

When continuing toward their expectations, most experience difficulties in imagining themselves using AI while aging. Developments are speeding and they are unsure whether they have to keep up with all these new developments. Will AI really take over? This is not an expectation most older adults visualize, but they think that AI influences more and more aspects in our lives. During the interviews, we listened to some music and discussed whether it was AI made or not. With close hearing, most could detect the more electronic sound in voices, but overall, it was hard to discover if music is made by AI. They enjoy our conversation and the things they learn in such a short time, which caused some arguing that it would be good to have courses or workshops on AI specific for the aging population to “keep it low key and understandable”, for example, provided by the library. However, not all were enthusiastic about the AI examples and expressed a desire for “the right to remain analogue” (Older Adult 7).

Three older adults consciously used AI; they used ChatGPT to answer questions—instead of using Google—to write a review about an event, to search for recipes, or to write a poem. They all were reserved at the start, but argue that ChatGPT replies often beyond their expectations:

I am familiar with AI, yes, I often use ChatGPT now instead of Google. I did not know what to expect from it, but I was intrigued to experience. The first time I tried ChatGPT was to write a poem for my grandson. It was a Sinterklaas poem to attach to the present. I understood that I had to provide ChatGPT with some information about my grandson and then ask it to write a poem. The poems I ask for are interesting, but still a bit mechanical. I had to rewrite some parts to make it more emotional and personal. But if you need the best recipe for apple pie it is very useful! (Older Adult 9)

I had to write a review about this festival in the harbour. My curiosity was already there since the introduction of ChatGPT, but did not try it out yet. Thus, I asked it to write a review about the festival. It actually provided a good review. The only thing I did not like, it was too slick. (Older Adult 13)

Interestingly, the people using/trying out AI are not those identifying themselves as innovators. The older adults who identified themselves as innovators mention that AI was something new and they do not work with it yet or even want to use it, mainly because they do not know what to expect from it. Furthermore, most assume that AI systems are not embedded in our society yet. Connected to this low degree of embedding, the idea that they will not be affected by AI in their lives is mainly expressed by the oldest participants. After the interviews, most turned a bit and had a more open view toward AI. It might not always be something with negative consequences, it contains more than they imagine, and there is already more in their lives than known without causing extreme reactions in society. Furthermore, some raise the question if we should always know whether a technology contains AI, and they expect that we do not always need to know this or need to understand it. This nicely flows into the discussion of XAI.

### Envisioning XAI

To follow their line of concerns, I continue the findings with older adults’ ideas toward explainability. I identified five views (see Figure 1), which are combined in three groups: (1) XAI is not desirable, (2) XAI is necessary, and (3) collaboration toward XAI.

#### XAI is not desirable

XAI is not desirable contains two views: (1) no—nothing, and (2) no—impossible. Starting with the first, there is just a small number who argue for *no—nothing*, thinking that XAI is not needed at all and AI should act “autonomous” to reach the best results. One of the participants with a mechanical technology background argues,

In the past we understood how all mechanics worked, but throughout time this became more and more difficult. On the one hand, I am too old to learn about AI, and on the other hand, I think that technologies will work best if we as humans do not interfere too much with them. (Older Adult 12)

This view is in accordance with the theory that AI can reach the best results if we do not consider explainability aspects, and do not need to diminish the functioning to remain understandable. In this theory, XAI can only be reached when we find a balance between technological capabilities and explainability, and search toward explanations diminishes the autonomy of an AI system.

Second, people who see themselves as unable to understand resemble the *no—impossible* group. The question, “Do I really want to know it all?” results often in a firm no. For example, by Older Adults 5 and 8. They do not only lack a desire toward explainability, but they argue that it is probably too difficult to learn and they do not have the capacity to understand. Although this is a more common view, not all participants who see themselves as incapable to learn and understand reject the idea of XAI. This group desires assistance, which is discussed in the next section. Getting back to the *no—impossible* group, there is a portion of older adults who do not see themselves interacting with an AI system to understand what it is doing and how it is doing things. They did not obtain knowledge about AI technologies during their lives, do not have the vision to be capable of obtaining the right knowledge in their remaining lives, and as Older Adult 16 clearly states, “it will not make sense to me at all.” Overall, this group is unsure about the need for XAI, but they do not fully reject XAI because maybe there are others who might show interest and see a value in explanations.

#### XAI is necessary

XAI is necessary includes the two following views: (3) yes—full and (4) yes—assisted. The participants who support the third view, *yes—full*, say that they need XAI because it is important to understand what happens and know details about the AI-made decisions in order to use decisions. Within this group, there are different views, ranging from the desire to understand all specifics and details, toward getting a basic understanding of the process toward a decision and what to do with it. Older Adult 14 is in favor of the latter:

You want to know where an outcome came from and what to do with it. So some kind of explanation is necessary, but not a mathematical approach. Most people have difficulties with math and we are just users of technology. At some point knowledge stops. If the system decides that I need to take certain medication or that I have a form of cancer, I would like to know how it forms this decision. Thus, ‘why’ does it make the decision and for example on what kind of information or knowledge is it based. It might also be useful to get information what I should do with a decision. (Older Adult 14)

Overall, the participants think that having XAI could assist in crossing the threshold toward using AI systems.

Older adults who are part of the fourth group, *yes—assisted*, need someone else to assist in order to reach understanding. So, they do not consider themselves as capable of understanding the XAI; therefore, they argue that XAI should be designed for those qualifying themselves as capable to understand. These people can share explanations with those who are not capable. “The aging population arrives in a digital world where they no longer have connections” (Older Adult 10). They see the need to understand what is happening and during the conversations they think that XAI could be an option to reach understanding for themselves with assistance, for example, from care professionals. Multiple participants are sorrowful about current changes in our society with which they cannot keep up with. They almost scream for help and assistance in dealing with digitalization and now AI makes this even more urgent. As part of this need for help is the aspect of meaningful communication with another person when AI becomes part of their aging lives. As an example, Older Adult 8 receives some home care, and she already uses digital portals to view files and results of tests. Although she thinks that these systems are valuable to cover first concerns, there is a need for human conversations coming with AI-made decisions or XAI:

Personal contact is valuable. With AI you don’t know for sure whether it’s all correct, you have to be able to check the results and it would be good to see if a lab technician agrees with findings from AI, for example. We must not lose touch and connection. Without explanations, chances are that we will all eventually stop thinking, become incapable to use personal experience, and lose knowledge. (Older Adult 8)

#### Collaboration toward XAI

Finally, view five, *yes/no—collaboration*, includes the older adults who acknowledge the need to know what happens within the AI, but also see the need to provide autonomy toward the AI system and have a kind of joint decision-making. This view has connections with the idea of human-in-the-loop and the importance of having a human element in the decision-making processes. A difference between human-in-the-loop and the fifth view is the emphasis put on collaboration within this view. Visions from my participants show the need to acquire knowledge on how a decision was established, and the option to ask questions to the AI system about the decision:

If it can show me how it works and can reply to my questions about a decision it will be easier to trust it fully or partially. (Older Adult 2)

Besides trust, also human touch and autonomy are important topics of the fifth view. Based on the ideas of the older adults, the envisioned future of the aging population with AI included both a human user and an AI system have autonomy in order to interact with each other. This means that the AI should be designed with little restrictions from humans, and the user’s autonomy could be reached with XAI. XAI could assist collaboration toward knowing and choosing what to take from an AI-made decision into a final decision. Older Adult 21 acknowledges that AI will be the future and we need this collaboration because

There are certain things that you notice differently as a human being. We are unique. … I understand that our healthcare system will incorporate AI and that we have to use it. In this future, I would like to know from my doctor what he thinks, what he takes away from the AI, and how he feels about it. For me it would be even more acceptable if the AI and the doctor both develop a diagnose or decision, and then compare differences or similarities. (Older Adult 21)

## Discussion

In this research, I explored what XAI is in the world of older adults while aging and how they imagine and value XAI by investigating their knowledge and visions regarding AI and XAI. The study showed that older adults imagine and value XAI in diverse manners. This insight is valuable for design and embedding in practice, and could be used in future research. It is in line with the theory on multiplicity by Mol (2002) because I was able to detect a multiplicity of XAI variants which were not set before, but made by the older adults. The findings of this research connect to the change in which XAI is slowly drifting outside the domain of computer science into the domain of social science. Taking the different realities of older adults together, I was able to determine five views (see Figure 1) that make sense to each older adult individually and together form a larger view on “what is XAI.”

A small group involved in my research claims that explanations, and therefore XAI, are not needed. This could be connected to ideas of secrecy, on which Seaver (2017) argues that secrecy might be something we should not be afraid of. The older adults provide different examples to underline their statement toward secrecy, like most people do not know what is happening within a car when we drive, but we keep on driving cars. If something seems to work and people see the benefit, they will use something with or without explanations. Taking this into the theory of sociotechnical systems, the black-boxed AI systems cause different enactments by creating certain social boundaries (Seaver, 2017). Secrecy in this perspective is an interesting social process enacted by insiders and outsiders and understanding is more than just knowing the relation between input and output of an AI system. Thus, some form of secrecy might not be harmful, which is in line with thoughts of most older adults, but although we do not know of most technologies how they work, most participants think that some form of understanding and lowering the secrecy of AI might be useful. For example, having full black boxes seems not desirable if a system provides a decision and the user cannot understand where it comes from or what to do with it. Furthermore, relating these findings to the CAT perspective (Peine & Neven, 2021), when AI gets introduced in the life-world of older adults it is now possible to study how they arise together. My study is a first step in studying how XAI and older adults arise together, and I found that there are not only different uses and expectations of AI, which are developing along with its introduction in our society, but there are also varying desires toward XAI and understandability. These desires toward XAI seem partly based on the capabilities and interest of older adults, but these might change within contexts and practices. Getting some form of information or being able to ask questions about a decision seems important before someone agrees, disagrees, or uses a decision. This is a valuable finding for future design of AI and the ideas around XAI.

Following on this, by embedding full black boxes in care practices, the AI system might create undesirable boundaries and reduce the place for expertise from care professionals (Seaver, 2017; Sendak et al., 2020). For a large number of the involved older adults, it seems important that an AI system, in the context of care, is not replacing care professionals. Therefore, when thinking about future directions for policy in care, explanations should be provided in a respectful way recognizing the expertise and capabilities of users (Shortliffe & Sepúlveda, 2018). This could be connected with the theoretically discussed triangular decision-making with XAI, care professionals, and patients (van Leersum & Maathuis, 2025). In the presented study, I empirically add that XAI could be desirable toward a kind of collaboration between AI and users, besides the already existing shared decision-making among humans. Thus, in the vision of older adults this means a form of decision-making in which each is equally recognized and can ask questions to form a joint understanding and decision.

However, with aging, AI is often taking toward the homes and lives outside care settings, asking for different explanations, possibly depending on the purpose of an AI system. Each AI system might ask for more or less explainability (Tielman et al., 2024), with the need for more or less assistance from others to understand and use, and the need for more or less joint decision-making processes. This will be an interesting aspect to explore further and connecting it further with co-constitution of aging and AI (Gallistl, Banday, et al., 2024). It would be interesting to understand how older adults live with AI and how XAI gets mutually shaped by each other. This is most in line with the collaborative XAI view, but further research seems crucial to explore how AI and XAI get contextualized in the lives of older adults.

The findings show some ideas toward this contextualization, mainly connected with an idea of Pesapane et al. (2024) to visualize AI-informed decision-making processes not as human-in-the-loop, but as algorithms-in-the-loop. Humans will close the circle and keep the algorithms-in-the-loop by asking questions and searching for explanations. In the findings, I present that the visions of older adults go beyond the human-in-the-loop view too, based on similar reasoning. Also Green and Chen (2019a, 2019b) argue in favor of the algorithm-in-the-loop because it is not only needed to involve humans into the process, but the users should have a possibility to interact with AI and explanations provided by the AI. They argue that XAI designed as algorithm-in-the-loop enhances a sociotechnical view by putting emphasis on social aspects rather than focusing on the technical context solely. Comparing this with research in the XAI domain, most focus on the technical aspects and raise a demand for uptake in social science. I took a first step and showed that for some there is a need to get back translating algorithms and outcomes (technical aspects), but for most it is more important to “collaborate” and get some context around an AI-made decision. In their view, the technical aspects are important, but how it is brought and become part of the more social aspects is crucial. This shows that the visions of older adults differ from the technological discourse because XAI is not only technical, but a constellation between humans and machines.

Thinking of explanations as this constellation, not all created models and explanation methods are reaching toward this. Focusing on the technical functionality of AI alone is not enough (Elish, 2018); combining social and technical understanding seems necessary to determine how XAI could be effective for the aging population. This idea should be taken in future design because having a full perspective of these social and technical aspects is as well important to create implementation processes that are ethically sound with recognition of bias and knowledge about possible disruptions of expertise and practices (Felber et al., 2025; Sendak et al., 2020). The application of AI in the context of aging asks for more careful consideration of ethical issues and possibilities for ageism (Gallistl, Banday, et al., 2024; Rubeis, 2020; van Leersum & Maathuis, 2025). The older adults did not discuss much about ethical issues, but some mentioned that AI might become an amplifier of existing age-related inequalities, which is an important finding to be taken by policy. By enhancing transparency with XAI, the explainability might assist in knowing and evaluating the age bias of a system. Thus, in view of the findings from my research, I agree with Tielman et al. (2024) who acknowledge that an AI system does not stand in itself and should be understood as a complex system in which different stakeholders will have different expectations, capabilities, and needs. I followed their advice to explore inclusive XAI by going beyond the visions of designers and developers, and talked with older adults as possible users.

### Strengths, limitations, and recommendations

I was able to interview 28 older adults, which is a strength of this study, showing the diversity of older adults in the Netherlands. In view of possible selection bias, based on the demographic characteristics (Table 1), I can reflect that I was able to reach a diversity in age, educational level, living conditions, and care needs. What could be improved is the relatively low number of older adults with a non-Dutch nationality. However, on a very positive note toward the aim of this study, even though I expected to reach more older adults with affinity toward technology, I was able to reach older adults with a variety of interest in technology and AI. Interesting to show was the normal distribution of innovators, adopters, and laggards within this group, in contrast to ideas about older adults as being technological laggards or late adopters. Although I used Rogers (2003) to question their position toward technology, I did not pose a similar question regarding their position toward AI. From the interviews, I can distinguish their use and view toward AI and interestingly these do not (always) correspond with their position toward technology. For example, a late adopter of technology was positive about AI and interested in use or an innovator of technology was a laggard when it comes to AI. Another aspect on which I should reflect is the lack of knowledge regarding the term XAI. Almost none of the included older adults knew about this before the interviews. This was expected and therefore I provided them with a definition which was used during the remaining parts of the interview. With this finding, it seems that older adults are less knowledgeable about (X)AI, but I wonder whether the same holds for younger adults, which would be interesting to explore and compare.

Further research into the feelings of being innovators versus being laggards to AI or being laggards versus early adopters of AI could be interesting. In the presented research, I have only talked with older adults, which did not provide me the possibility to explore relationships with and enactments of AI, for example, by including family caregivers. Future research including ethnographic research within communities/associations of older adults might be assistive in exploring relationships and enactments of AI in the lives and practices of older adults. Furthermore, to deepen our understanding of the perspectives of XAI, it might be interesting to perform research in residential care settings. Now it was possible to explore the visions of older adults in general, but less on care specific topics. Future research could add the layer of care as well as family caregivers. I did not include them actively in the presented study, but their role in the lives of older adults is crucial and they will be affected by AI as well. Probably they can have a role in assisting with understanding XAI when requested by the older adults. Also, following on from the presented findings, as a future research step in the field of XAI in care for older adults it seems required to identify the impact of a specific AI system in the context of use (Tielman et al., 2024). Then, it will be possible to question explainability in practice, observe the interaction between older adults and AI, and investigate the enactment of AI that is explainable in an inclusive way by involving different stakeholders and using participatory approaches to design the explanations.

Other recommendations are connected to the concern of little research on inclusive XAI (Tielman et al., 2024). It is recommended to investigate how explainable a system should be for specific user populations, such as the aging population, to determine how we should craft explanations matching with capabilities of users, and how we can achieve these explanations. The presented study contributes to the first and partly to the second question, but future participatory research seems needed to investigate all questions and create further understanding of inclusive XAI in the context of aging. It would be interesting to perform co-creation sessions with older adults to creatively assist them in developing views regarding their own life in our digital society. These sessions can assist in having a more situated understanding how they envision and place (X)AI in their lives.

When taking a focus further toward providing explanations and creating them in participatory research, it might be possible to divide XAI in categories, such as explanations that enhance understanding of the algorithm, or explanations that enhance understanding of a decision or outcome provided by the machine using the algorithm (Timmer et al., 2017). I think both are important, but my research mainly focused on the latter. Butz et al. (2022) made some recommendations toward explanatory elements in XAI focusing on the understanding of an AI informed decision-making process. They argue that a combination of visualizations and simple text will help most individuals, and they think that more precise explanations will be perceived as useful compared with short causal chains (Butz et al., 2022). These ideas are interesting to incorporate in my suggestion toward participatory research in which older adults, and other stakeholders are actively collaborating to develop these kinds of explanations and XAI in actual practice. Although Butz et al. (2022) argue that this kind of research is too time-consuming, I think it will be the most fruitful way of future research, also in line with Miller (2019a) who argues that we need to learn from the actual users how they form and give explanations, and how these “human” explanations are received by others. Participatory research approaches will then be most suitable to further develop and explore XAI with the aging population inside and outside care settings.

## Conclusion

With an expanding number of older adults living independently, technologies are said to bring support in performing daily practices. AI, for example, in smart sensors or conversational agents, is becoming part of our lives and has the potential to improve aging in place. XAI might assist in opening opaque black boxes and enhance understanding of the underlying logic of an AI-made decision. This study shows that the knowledge on AI varies, and although most are not intending to use it, there are some who show interest and see a positive future with AI. The older adults express different values and needs toward explainability, leading to five views toward XAI in which XAI is not desirable, XAI is necessary, or collaboration is preferred. XAI is different from the current technological discourses because it is not only technological, but a constellation between humans and machines, within which most older adults prefer having a kind of joint decision-making process.

## Supplementary Material

gnaf308_Supplementary_Data

## Data Availability

All relevant data are in the manuscript and its supporting materials. The datasets that support the findings and conclusion of this study are available from the corresponding author on reasonable request. The data are not publicly available due to privacy and/or ethical restrictions.
