# Supplementary material for: What should AI explain to you? Visions of older adults regarding explainable AI
Source: Gerontologist. 2026 Feb 3;66(2):gnaf308. doi: 10.1093/geront/gnaf308 (PMC13135710; doi:10.1093/geront/gnaf308)
Supplement: gnaf308_Supplementary_Data [file gnaf308_supplementary_data.pdf]

Supplementary material 1: Interview protocol explainable AI according to older adults

Opening

Briefly introduce myself, purpose of the research, research and purpose of interview, refer to information letter and consent form and then ask if recording may be started.

Questions about technology in general and their lives/health

- Can you briefly tell something about yourself?
- What technologies do you use in your daily life?
  - At what times? In what way?
  - Has it changed anything in your daily activities?
  - What was the reason for using this technology?
  - Is the technology a replacement for something else?
- Do you use technology for your health?
  - If yes: Can you tell more about this technology? What kind of technology, how do you use the devices, what are your experiences?
  - If yes: How satisfied are you with this technology?
  - If yes: Is there artificial intelligence in this technology? Do you know how this artificial intelligence works?
  - If no: What are reasons for you not to use technology?
- Do you understand how to use the technology?
  - How did you learn this?
  - Do you need assistance to use technology? If yes: What kind of assistance and from whom?
- What do you need to be able to use technology well?
- Do you think technology has added value?
  - Can you explain this answer further?

Questions about artificial intelligence

- Have you heard about artificial intelligence?
  - If yes: Can you tell me what you know about artificial intelligence?
  - If yes: Where did acquire knowledge about artificial intelligence?
  - If no: The researcher will give some examples and questions whether the participant knows about these developments.
- Do you understand how artificial intelligence works?
- What information do you need to use artificial intelligence?
- How would you evaluate the influence of artificial intelligence in our society?

Questions about the data collected by technology

- Are you aware of data collection by technologies? Can you elaborate on that?

    - If yes: What kind of actions do you take regarding data collection?
- When you use technology (possibly take an example earlier mentioned by the participant), what data do you see?
    - Do you know what data the technology can collect?
    - Do you know what happens to this data?
    - How do you deal with this data?
- Do you think data collection by technology is a chance or a threat in our society? Can you elaborate on that?

Questions about the explainability of AI

- Are you familiar with the term explainable AI?
    - If yes: Can you explain in your own words how you understand this concept?
    - If no: The researcher will provide an explanation of the concept.
- How do you think explainable AI should take shape?
- Do you think explainable AI is needed in healthcare or is the outcome of a device sufficient? Can you elaborate on that?

Questions related to the need for explainability

- What are the relevant skills for you to use AI?
    - Do you have these skills or are there others who need to assist you?
- What level of understanding is needed to understand AI?
    - What knowledge do you need to find AI reliable?
    - What do you want to know about the operation or outcome of a device?
    - Could you make decisions using AI? In what way?
    - How would explainable AI help you use AI?
    - What knowledge do you need to make decisions using AI?
    - What do you need to understand the use of AI?

If you look back at the conversation: What are your wishes regarding explainable AI and its use in your life and possibly care?

Closing

All questions answered now, a few more short questions to conclude and for follow-up.

- Would you recommend someone I can ask for an interview?
- Do you have any questions for me?
- If we have any further questions, can I contact you at a later time?

Questions some demographics if not discussed during the interview

- Age
- Education level
- Living conditions
- Work
- Family
- Nationality
- Care needs/health
- Type innovator

Thank you for your time.

The researcher will tell them that they will share the findings of the interviews and ask if anyone would like to be involved in future studies.
